# Understanding exacerbation risk in BLVR: A logistic regression approach to complication prediction

**DOI:** 10.1177/14799731261443319

**Published:** 2026-04-22

**Authors:** Johannes Wienker, Kaid Darwiche, Rüdiger Karpf-Wissel, Erik Büscher, Johannes Haubold, David Kersting, Hubertus Hautzel, Lukas van de Sand, Aymen Kassem, Kristin Mersmann, Christian Taube, Marcel Opitz, Marc Struß

**Affiliations:** 1 Department of Pulmonary Medicine, Division of Interventional Pneumology, University Medicine Essen-Ruhrlandklinik, Essen, Germany; 2 Institute of Diagnostic and Interventional Radiology and Neuroradiology, 39081 University Hospital Essen, Essen, Germany; 3 Department of Nuclear Medicine, 39081 University Hospital Essen, Essen, Germany; 4 Department of Infectious Diseases, West German Centre of Infectious Diseases, 39081 University Hospital Essen, Essen, Germany; 5 Department of Emergency Medicine, 39081 University Hospital Essen, Essen, Germany; 6 Department of Pulmonary Medicine, University Medicine Essen-Ruhrlandklinik, Essen, Germany

**Keywords:** chronic obstructive pulmonary disease, emphysema, bronchoscopic lung volume reduction, valves, pneumonia, exacerbation, infectious complications

## Abstract

**Background:**

Chronic obstructive pulmonary disease (COPD) with emphysema is associated with persistent airflow limitation and frequent exacerbations. Bronchoscopic lung volume reduction (BLVR) with endobronchial valves (EBVs) improves lung function and quality of life but carries a risk of postprocedural complications, including acute exacerbations and pneumonia. Predictors of these adverse events remain incompletely defined.

**Purpose:**

To identify clinical and inflammatory factors associated with postprocedural exacerbations in patients undergoing BLVR with EBVs, aiming to support individualized risk stratification.

**Patients and Methods:**

We retrospectively analyzed 320 patients with advanced emphysema treated with EBVs between 2015 and 2022. Patients underwent comprehensive preprocedural evaluation, including pulmonary function testing, imaging, perfusion scintigraphy, 6-minute walk test and COPD Assessment Test. Postprocedural exacerbations within 8 weeks were documented clinically and radiographically. Binary logistic regression, including multivariable modeling, was used to identify independent predictors.

**Results:**

Thirty-five patients (10.9%) developed post-BLVR exacerbations, six of whom had pneumonia. Exacerbation risk was independently associated with diabetes mellitus type II (OR 11.0, *p* < 0.001), elevated C-reactive protein >1 mg/dL (OR 9.35, *p* < 0.001), WBC >11 cells/nL (OR 5.46, *p* = 0.002), prior exacerbation frequency (OR 2.94, *p* < 0.001) and low BMI (OR 0.78, *p* < 0.001). Residual volume showed a trend toward significance (*p* = 0.058). The final model demonstrated excellent discriminative ability (AUC = 0.923). While lung function improvement was attenuated in the exacerbation group, quality of life gains were comparable.

**Conclusion:**

Elevated inflammatory markers, diabetes, frequent prior exacerbations and low BMI were independently associated with early postprocedural exacerbations following BLVR with EBVs. Comprehensive preprocedural assessment and targeted management of these risk factors may enhance patient safety and improve outcomes.

## Introduction

Chronic obstructive pulmonary disease (COPD), including emphysema, is a major global health concern, primarily driven by tobacco exposure and characterized by persistent respiratory symptoms and airflow limitation. Among patients with advanced emphysema, bronchoscopic lung volume reduction (BLVR) using endobronchial valves (EBVs) has emerged as an established minimally invasive treatment option, offering improvements in lung function, exercise capacity and quality of life.^1–3^

Despite the demonstrated benefits of BLVR, the procedure is not without risks. Post-procedural complications such as acute exacerbations (AECOPD), pneumonia and pneumothorax are relatively common and may significantly impact patient outcomes, recovery time and healthcare utilization.^4,5^ These complications can occur both in the peri-interventional period and in the first months following valve implantation. However, the underlying risk factors predisposing patients to these adverse events remain insufficiently characterized.

Identifying reliable predictors of peri- and post-interventional exacerbations and pneumonia is essential for improving patient selection, optimizing pre- and post-procedural care and ultimately enhancing the safety and efficacy of BLVR. While previous studies have investigated efficacy predictors and the overall safety profile of EBV treatment, few have focused specifically on the predictors of these clinically relevant complications.^6–8^

In this project, we aim to identify potential factors associated with an increased risk of procedure associated inflammatory events in emphysema patients undergoing BLVR with endobronchial valves. Our goal is to support individualized risk stratification strategies to enhance clinical decision-making and improve patient outcomes.

## Methods

### Patients

#### Study population

In this retrospective single-center study, we evaluated patients with advanced emphysema who underwent BLVR with one-way endobronchial valves (Zephyr©; PulmonX, CA, USA) between January 2015 and December 2022. Candidates were considered after exhaustion of optimal medical and supportive therapy and were required to have a forced expiratory volume in one second (FEV_1_) <45% predicted and a residual volume (RV) >180% predicted. Patients with more than three exacerbations per year were temporarily excluded until clinical stabilization. In selected cases, minor deviations from these thresholds were tolerated.

Preprocedural evaluation included pulmonary function testing, perfusion scintigraphy, fissure analysis with quantitative computed tomography and Chartis (catheter-based; PulmonX) echocardiography, a 6-minute walk test, and assessment of health-related quality of life using the COPD Assessment Test. All cases were reviewed by a multidisciplinary board comprising interventional pulmonologists, thoracic surgeons and radiologists.

All patients completed pulmonary rehabilitation, typically within six months preceding BLVR and abstained from smoking for at least six months prior to BLVR, verified by normal serum carboxyhemoglobin and urinary cotinine levels. Following valve implantation, patients were monitored in-hospital for a minimum of four days to allow early detection of complications, particularly pneumothorax. Peri-interventional antibiotic prophylaxis with ampicillin was administered for three days according to institutional protocol. Follow-up assessments, including pulmonary function testing, 6-minute walk test and CAT score, were performed three months after the procedure.

The study was conducted in compliance with the guidelines of the Institutional Review Board of the University Hospital Essen. The Ethics Committee of the University of Duisburg-Essen (Approval Number 24-12650-BO) waived the informed consent due to the retrospective and anonymous nature of this study. The data were completely anonymized before being included in the study.

### Outcome variables

Patients were divided into two main groups: those who experienced an AECOPD during or after the intervention and those who did not develop acute inflammatory events. An AECOPD was defined clinically by worsening respiratory symptoms requiring treatment with antibiotics, systemic corticosteroids or a combination of both. Pneumonia was defined separately by the presence of a new pulmonary infiltrate on imaging in combination with clinical signs of infection. Due to the retrospective design and potential overlap between both entities, these events were analyzed together as postprocedural inflammatory events, while pneumonia cases were reported descriptively. The 8-week time window was chosen to capture early peri- and post-interventional complications, which are most likely to occur shortly after BLVR, in line with prior studies and clinical practice.^5^

For the purpose of this study, the peri-interventional period was defined as the time from the procedure up to hospital discharge (typically within 4–5 days), while the post-interventional period referred to the time after discharge up to 8 weeks following BLVR.

### Statistics

Binary logistic regression was used to identify predictors of post-BLVR exacerbation and pneumonia. Variables were first analyzed blockwise using backward stepwise regression and significant predictors were then entered into a final multivariable model using the Enter method. Model fit was assessed with the Hosmer–Lemeshow test and pseudo R^2^ values, while discriminative ability was evaluated using ROC curve analysis and the area under the curve (AUC). Multicollinearity was checked via tolerance and variance inflation factor (VIF). Variables were subjected to the Shapiro–Wilk test to determine non-normal distribution. For CRP and WBC, clinically established cut-off values were used to facilitate interpretability and clinical applicability of the regression model. Sensitivity analyses using continuous variables yielded comparable results (data not shown). Two-group differences were assessed using an independent-samples t-test for parametric data or the Mann–Whitney test for non-parametric data and categorical data were compared using the chi-squared test. A *p* value < 0.05 was considered statistically significant. All statistical analyses were conducted using SPSS version 23 (IBM, New York, NY, USA).

## Results

**Figure 1.**
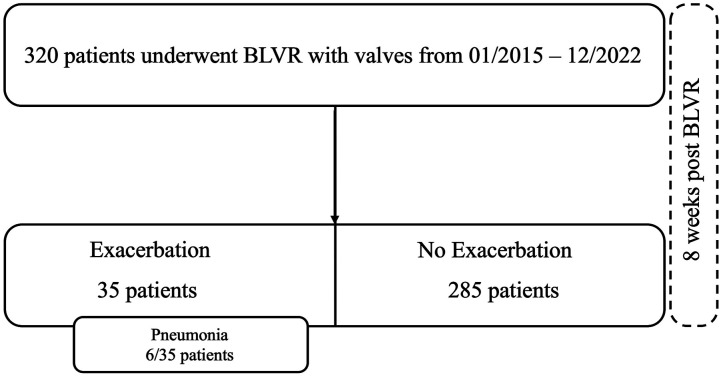
Data availability flow chart. BLVR, bronchoscopic lung volume reduction.

A total of 320 patients undergoing BLVR with endobronchial valves with complete data were included in the analysis. Among these, 35 patients (10.9%) experienced a postprocedural exacerbation within 8 weeks of the procedure, while 285 (89.1%) did not. Notably, 6 of the 35 patients with exacerbation were diagnosed with pneumonia, either concurrently with the exacerbation or as a direct consequence of it (Figure 1). Baseline demographic and clinical characteristics are summarized in Table 1.

**Table 1.** Demographic and clinical characteristics at baseline (pre BLVR).

Parameter	Median (range) or number (%)		P value
	Exacerbation	No Exacerbation	
Female/male, n	30/5	107/178	**0.008**
Age, years	56.3 (38-76)	57.8 (40-82)	0.160
Body mass index, kg/m^2^	18 (15 – 30)	22.6 (14 – 36)	**<0.001**
Smoking history, pack-years	32.5 (25 – 90)	35 (30 – 90)	0.222
CAD/HF	11 (31)	88 (31)	0.883
DM II	21 (60)	29 (10)	**<0.001**
CKD	2 (6)	7 (2)	0.257
Exacerbations			
0	6 (17)	154 (54)	**<0.001**
1	9 (26)	71 (25)	
2	9 (26)	37 (13)	
3	11 (31)	23 (8)	
CRP, mg/dL	1.1 (0.1 – 5)	0.5 (0 – 2.4)	**<0.001**
WBC, cells/nl	12 (5 – 17)	8.3 (0.4 – 8.3)	**<0.001**
FEV_1_			
Liters	0.81 (0.44– 1.60)	0.75 (0.3– 1.44)	0.216
% predicted	25 (13 – 46)	27 (8 – 47)	0.832
RV			
Liters	5.9 (3.7 – 11.4)	5.6 (3.2 – 11.6)	**0.013**
% predicted	270 (204 – 464)	260 (163 – 474)	0.182
IVC			
Liters	2.6 (1 – 4.5)	2.21 (0.6 – 4.6)	0.143
% predicted	69 (37 – 107)	67 (13 – 112)	0.886
CAT score	27.5	26	0.235
6MWT, m	256 (120 – 453)	287 (95 – 520)	0.061

Values are Median (Range) or Number (%).

FEV_1_, forced expiratory volume in 1 second; RV, residual volume; BLVR, bronchoscopic lung volume reduction, CAD/HF, Coronary artery disease or heart failure; DM II, diabetes mellitus type 2; CKD, chronic kidney disease; CRP, C-reactive protein; WBC, white blood cell count; 6MWT, 6 Minute Walk Test.

Patients with postprocedural exacerbations were more likely to be female (*p* = 0.008), had a significantly lower body mass index (BMI, median 22.6 vs. 18.0 kg/m^2^, *p* < 0.001) and a higher prevalence of type 2 diabetes mellitus (DM II, 60% vs. 10%, *p* < 0.001). Inflammatory markers were markedly elevated in the exacerbation group, including higher C-reactive protein (CRP) levels (median 1.1 vs. 0.5 mg/dL, *p* < 0.001) and white blood cell (WBC) counts (median 8.3 vs. 12 cells/nL, *p* < 0.001). The CAT scores were 27.5 in the exacerbation group and 26.0 in the non-exacerbation group, with no statistically significant difference between groups (*p* = 0.235). Exercise capacity as assessed by the 6-minute walk test (6MWT) did not differ significantly between patients with and without postprocedural exacerbations and was not associated with exacerbation risk.

Patients who later developed exacerbations also reported a higher number of COPD exacerbations per year prior to BLVR (*p* < 0.001) and demonstrated higher baseline RV in liters (median 5.9 vs. 5.6 L, p = 0.013). No significant differences were observed in age, smoking history or FEV_1_ at baseline.

All acute exacerbation events were documented within an 8-week period following the intervention, encompassing both peri-interventional (Four to five days during hospitalization) and early post-interventional phases. Among 35 patients with AECOPD, 32 required systemic antibiotic therapy, systemic corticosteroids and intensified inhalation therapy. In the remaining 3 patients, symptoms were mild enough to permit outpatient corticosteroid therapy. Bronchoscopy was performed in 28 cases to evaluate potential valve migration and to obtain microbiological specimens. Microbiological analysis identified pathogenic bacterial infections in 24 cases. In 6 patients, a concomitant or subsequent pneumonia was diagnosed, confirmed by corresponding pneumonic infiltrates. Microbiological results are provided in Supplemental Table S1.

Follow-up lung function was measured at 3 months post-BLVR. Both groups showed improvements in lung function parameters, but the magnitude of change was significantly smaller in the exacerbation group and the percentage change in FEV_1_ was negative in the exacerbation group (–1.9%) compared to a 13% improvement in the non-exacerbation group (*p* < 0.001). Differences in changes of RV and IVC were not statistically significant. The CAT scores improved to 25.5 in the exacerbation group and 24 in the non-exacerbation group, again without a significant between-group difference (*p* = 0.267). The change in CAT score from baseline to follow-up was comparable in both groups (*p* = 0.313), indicating that both cohorts experienced a similar improvement after the intervention. Complete lobar atelectasis occurred at similar rates in both groups (*p* = 0.584). Achievement of the minimal clinically important difference (MCID) of 563 mL for RV reduction was seen in 31 patients (88%) with exacerbation and 220 patients (77%) without exacerbation, which was also not statistically significant (*p* = 0.189) (Table 2).^9^

**Table 2.** Clinical characteristics post BLVR.

Parameter	Median (range) or number (%)		P value
	Exacerbation	No Exacerbation	
FEV_1_			
Liters	0.77 (0.40– 1.40)	0.85 (0.38– 1.75)	0.255
% predicted	28 (13 – 54)	31 (15 – 66)	0.278
% change	– 1.9 (-20 – 46)	13 (-42 – 184)	**<0.001**
RV			
Liters	5.1 (3.2 – 8)	4.9 (2.9 – 12.3)	0.053
% predicted	232 (124 – 389)	222 (22 – 642)	0.221
% change	– 15.1 (-55 – 25)	– 14.3 (-56 – 92)	0.324
IVC			
Liters	2.8 (1.5 – 4.9)	2.5 (1.1 – 4.8)	0.190
% predicted	72 (45 – 106)	76 (35 – 139)	0.908
% change	12.7 (-49 – 282)	9.2 (-28 – 80)	0.488
CAT			
Score	25.5	24	0.267
Change in Score	- 2	- 2	0.313
6MWT			
Meters	322	305	0.336
Change in Meters	35	49	0.276
Complete Atelectasis	18 (51)	166 (58)	0.584
RV-MCID	31 (88)	220 (77)	0.189

Values are Median (Range) or Number (%).

FEV_1_, forced expiratory volume in 1 second; RV, residual volume; BLVR, bronchoscopic lung volume reduction, CAD/HF, Coronary artery disease or heart failure; DM II, diabetes mellitus type 2; CKD, chronic kidney disease; CRP, C-reactive protein; WBC, white blood cell count; RV-MCID, minimally clinically important difference for RV = 563 mL; 6MWT, 6 Minute Walk Test.

A multivariable binary logistic regression analysis was performed to identify independent predictors of post-interventional exacerbation following BLVR with EBVs (Table 3). All variables that were found to be significant in previous blockwise backward-elimination models (including comorbidities, baseline pulmonary function, laboratory markers and procedural characteristics) were entered into a final model.

**Table 3.** Blockwise Logistic Regression model for inflammatory complication in BLVR patients.

​	Odds ratio	95% CI	P value
Clinical characteristics – excluded Age, Gender, Pack Years			
Age _excl._	0.968	0.925 - 1.013	0.156
Gender _excl._	2.341	0.851 – 6.439	0.099
Pack years _excl._	1.013	0.994 – 1.032	0.190
**BMI**	**0.808**	**0.721 – 0.906**	**<0.001**
Comorbidities – excluded CAD/HF, CKD			
CAD/HF _excl._	1.106	0.535 – 2.285	0.786
**DM II**	**14.105**	**5.143 – 38.686**	**<0.001**
CKD _excl._	1.244	0.065 – 23.645	0.885
**Exacerbations/yr (0 – 3)**	**2.341**	**1.468 – 3.735**	**<0.001**
Laboratory parameters – excluded GFR, Hemoglobin, Thrombocytes			
**CRP >1mg/dL**	**4.845**	**2.220 – 10.574**	**<0.001**
**WBC >11 cells/nL**	**5.241**	**2.345 – 11.714**	**<0.001**
GFR <60 ml/min/1.73 m^2^_excl._	0.423	0.069 – 2.603	0.354
HgB <10g/dL _excl._	0.940	0.722 – 1.224	0.646
TC >300 cells/nL _excl._	0.994	0.988– 1.001	0.075
Baseline respiratory parameters – excluded FEV_1_, IVC, DLCO, LTOT			
FEV_1_ L _excl._	5.428	0.336 – 87.715	0.233
**RV L**	**1.607**	**1.175 – 2.198**	**0.003**
IVC L _excl._	1.104	0.501 – 2.434	0.806
TLCO % _excl._	0.998	0.949 – 1.050	0.938
LTOT (vs. no LTOT) _excl._	2.425	0.885 – 6.642	0.085
Procedural parameters – excluded Nr. valves, side, pneumothorax, atelectasis			
Nr. valves (2-8) _excl._	1.109	0.748 – 1.645	0.607
Side (left vs. right) _excl._	1.456	0.424 – 4.995	0.551
Pneumothorax _excl._	2.074	0.905 – 4.754	0.085
Atelectasis _excl._	0.748	0.357 – 1.570	0.443

BMI, body mass index; CAD, coronary artery disease; HF, heart failure; DM, diabetes mellitus; CKD, chronic kidney disease; CRP, c-reactive protein; WBC, white blood cell count; GFR, glomerular filtration rate (CKD-EPI-equation); HgB, hemoglobin; TC, thrombocyte count; FEV1, forced expiratory volume in the first second; RV, residual volume; IVC, inspiratory vital capacity; TLCO, factor of the lung for carbon monoxide; LTOT, long term oxygen therapy; Statistical significance marked bold.

The model was statistically significant (Omnibus Test of Model Coefficients: χ^2^ = 110.85, df = 6, *p* < 0.001), indicating that the predictors as a set contributed significantly to the outcome. The model explained approximately 58.7% of the variance in post-BLVR exacerbation, as indicated by Nagelkerke’s R^2^ = 0.587. The Hosmer-Lemeshow goodness-of-fit test showed no evidence of poor model fit (χ^2^ = 12.88, df = 8, *p* = 0.116).

To assess the model’s discriminative ability, a ROC curve analysis was performed using the predicted probabilities from the regression model. The analysis yielded an AUC of 0.923, indicating excellent accuracy in distinguishing between patients who did and did not develop an exacerbation following BLVR.

In the final model (Table 4), higher BMI was independently associated with a lower risk of exacerbation (OR = 0.784; 95% CI: 0.689–0.893; *p* < 0.001), indicating a protective effect. In contrast, the presence of diabetes mellitus type II significantly increased the odds of exacerbation (OR = 11.006; 95% CI: 3.747–32.322; *p* < 0.001). Likewise, a greater number of COPD exacerbations in the year prior to BLVR was associated with increased risk (OR = 2.941; 95% CI: 1.752–4.935; *p* < 0.001). Elevated inflammatory markers also showed strong associations: patients with CRP levels >1 mg/dL had markedly higher odds of exacerbation (OR = 9.346; 95% CI: 3.023–28.895; *p* < 0.001), as did those with WBC counts >11 cells/nL (OR = 5.462; 95% CI: 1.854–16.095; p = 0.002). Baseline RV in liters demonstrated a trend toward statistical significance (OR = 1.500; 95% CI: 0.986–2.282; *p* = 0.058). Pneumothorax occurred in 51 cases (16%, 42 versus 9 for No Exacerbation versus Exacerbation) and was not independently associated with postprocedural exacerbations risk.

**Table 4.** Final Regression model for inflammatory complication in BLVR patients.

​	Odds ratio	95% CI	p-value
Clinical characteristics – excluded RV			
**BMI**	**0.784**	**0.689 – 0.893**	**<0.001**
**DM II**	**11.006**	**3.747 – 32.322**	**<0.001**
**Exacerbations/yr (0 – 3)**	**2.941**	**1.752 – 4.935**	**<0.001**
**CRP >1mg/dL**	**9.346**	**3.023 – 28.895**	**<0.001**
**WBC >11/nL**	**5.462**	**1.854 – 16.095**	**0.002**
RV L	1.500	0.986 – 2.282	0.058

BMI, body mass index; DM, diabetes mellitus; CRP, c-reactive protein; WBC, white blood cell count; RV, residual volume. Statistical significance marked bold.

## Discussion

In this retrospective analysis of patients undergoing BLVR, several clinical and inflammatory factors were identified as independently associated with postprocedural exacerbations. The results provide novel insight into the complex interplay between baseline patient characteristics, systemic inflammation and procedural outcomes in a population with advanced emphysema.

Our findings demonstrate that elevated CRP (>1 mg/dL) and WBC (>11 cells/nL) were among the strongest independent predictors of postprocedural exacerbations. This is consistent with previous studies that have linked systemic inflammation to exacerbation risk and poor outcomes in COPD. For example, Agustí et al. found that elevated CRP was associated with increased frequency of exacerbations and worse prognosis, even in clinically stable patients.^10^ Similarly, Sapey et al. showed that neutrophilic inflammation in the airways correlates with systemic WBC elevation and predicts future exacerbations.^11^

These inflammatory markers likely reflect an underlying state of heightened immune activation, either due to chronic infection, immune dysregulation or subclinical inflammation in the lungs. In the context of BLVR, where procedural manipulation may transiently disturb airway integrity or mucus clearance, patients with elevated inflammatory markers may be more susceptible to infective or non-infective exacerbations triggered by the intervention itself.^12,13^ Our findings suggest that preprocedural inflammatory status should be considered when assessing candidacy and risk stratification for BLVR.

The presence of DM II was associated with an approximately 11-fold increased risk of postprocedural exacerbation, making it one of the most powerful predictors in our model. This aligns with prior evidence linking metabolic comorbidities to increased susceptibility to infections and systemic inflammation in COPD. Diabetic patients often exhibit impaired neutrophil function, reduced mucociliary clearance and altered lung microbiota, which may contribute to increased vulnerability post-intervention.^14^ Additionally, diabetes is associated with higher baseline CRP and WBC levels, which may further exacerbate the inflammatory milieu.^15^

A study by Cazzola et al. also reported that COPD patients with diabetes experience more frequent exacerbations and longer recovery times, particularly when systemic corticosteroids are used. In the context of BLVR, this may translate into a higher likelihood of postprocedural complications and infections.^16^ These results underscore the need for careful glycemic control and possibly tailored perioperative management strategies in diabetic patients considered for valve therapy.

Interestingly, our analysis found that higher body mass index (BMI) was inversely associated with exacerbation risk. This finding supports the so-called “obesity paradox” in COPD, where overweight and mildly obese patients often demonstrate better clinical outcomes than their underweight counterparts.^17^ Low BMI is frequently a marker of advanced disease, malnutrition and systemic catabolism in COPD and has been associated with poor treatment response and increased mortality.^18^

In the context of BLVR, patients with higher BMI may have better respiratory muscle reserves and improved immune function, which could contribute to a lower risk of postprocedural exacerbations. Moreover, cachectic patients may exhibit poorer wound healing and immune surveillance following invasive procedures.^19^ Our results reinforce the prognostic relevance of BMI and suggest that patients with low BMI may require closer monitoring and nutritional support during the peri-interventional period.

In our cohort, both patients with and without postprocedural exacerbations demonstrated modest but clinically significant improvements in CAT scores following BLVR. No significant differences between groups were observed either at baseline, at follow-up or in the magnitude of score change. These findings suggest that, despite the occurrence of exacerbations in the early postprocedural period, patients may still achieve symptomatic benefit as perceived by patient-reported outcomes. This is consistent with previous studies showing that the symptomatic effects of BLVR can be maintained even in the presence of certain postprocedural complications, provided these are effectively managed.^1,6^ The absence of a significant difference between groups also highlights that exacerbations, while impacting lung function recovery in some patients, do not necessarily negate gains in patient-reported quality of life. Nonetheless, the improvements in CAT scores were relatively small and did not reach statistical significance within or between groups, underscoring that changes in health status after BLVR are multifactorial and may be influenced by baseline disease severity, comorbidities and patient expectations.

The number of COPD exacerbations in the year preceding BLVR was a strong and consistent predictor of postprocedural events. This finding is in line with the well-established “frequent exacerbator” phenotype described in numerous studies, including the ECLIPSE trial, where patients with ≥2 exacerbations per year had a higher risk of future events regardless of baseline lung function.^20,21^ This phenotype likely reflects a combination of host susceptibility (e.g., bacterial colonization, airway hyperresponsiveness) and persistent inflammation.

The inclusion of exacerbation frequency in our model emphasizes the need for thorough longitudinal assessment before BLVR. While BLVR aims to improve airflow and reduce hyperinflation, it may not directly modify the intrinsic tendency for exacerbations in certain patients. Thus, identifying frequent exacerbators prior to the procedure could help guide decisions on further prophylactic antibiotic use, close follow-up or alternative treatment strategies.

RV demonstrated a trend toward significance in the final model (*p* = 0.058), suggesting a possible association between greater hyperinflation and exacerbation risk. RV is a key marker of air trapping and dynamic hyperinflation, both of which are linked to dyspnea and impaired clearance of secretions. Previous studies have reported that greater baseline RV is associated with increased risk of exacerbations and worse health status.^22,23^ While RV is also one of the main indicators for BLVR candidacy, our findings suggest that in patients with particularly high hyperinflation, careful postprocedural monitoring is warranted, as they may be more vulnerable to postprocedural destabilization.

In our cohort, the proportion of patients achieving complete lobar atelectasis following BLVR did not differ significantly between those with and without postprocedural exacerbations (51% vs. 58%, *p* = 0.584). This aligns with multicenter data indicating that complications do not necessarily diminish the clinical benefits of BLVR, particularly when target lobe collapse is achieved. A large retrospective study found that clinical outcomes, such as lung function and quality of life, were comparable in patients with and without atelectasis, despite procedural complications.^24^

The microbiological spectrum observed in our cohort underscores the heterogeneity of bacterial pathogens in post-BLVR respiratory events. In patients with AECOPD, Gram-negative pathogens were predominant, including *Escherichia coli*, *Klebsiella pneumoniae*, *Proteus mirabilis*, *Serratia marcescens* and *Citrobacter koseri*, alongside common COPD-associated organisms such as *Moraxella catarrhalis* and *Pseudomonas aeruginosa*. Gram-positive bacteria were less frequently isolated, with *Staphylococcus aureus* identified in three cases. Notably, patients presenting with pneumonia demonstrated a narrower pathogen range, with *Pseudomonas aeruginosa* and *Streptococcus pneumoniae* being the most frequent, alongside occasional *Klebsiella pneumoniae* and *Proteus mirabilis*.

This distribution differs somewhat from the typical bacterial profile of community-acquired pneumonia in COPD, where *Streptococcus pneumoniae*, *Haemophilus influenzae* and *Moraxella catarrhalis* predominate.^25,26^ The higher representation of Enterobacteriaceae and non-fermenting Gram-negatives in our AECOPD group may likely reflect the impact of advanced disease stage, frequent prior antibiotic exposure and possible colonization patterns associated with severe emphysema and repeated hospitalizations. Importantly, the isolation of *Pseudomonas aeruginosa* in both AECOPD and pneumonia cases is clinically relevant, as its presence has been linked to worse outcomes, prolonged hospitalization and the need for targeted antimicrobial therapy in COPD exacerbations.^27^

The results of this study have several important implications for clinical practice. First, inflammatory markers (CRP, WBC) and diabetes mellitus should be considered in preprocedural risk stratification for patients undergoing BLVR. These variables are readily available and may help identify high-risk patients who could benefit from intensified clinical and laboratory monitoring or alternative treatment strategies.

Second, our findings highlight the value of assessing prior exacerbation history and nutritional status (BMI) when evaluating candidacy for valve therapy. Interdisciplinary management including optimal nutrition, pharmacology, training and respiratory physiotherapy may help optimize modifiable risk factors and improve outcomes.

Finally, future research should aim to develop a validated risk integrating clinical, inflammatory and functional variables to predict postprocedural exacerbation risk and tailor patient selection accordingly.

This study has several limitations. It is retrospective in nature, which limits causal inference and conducted at a single emphysema care center. The definition of postprocedural exacerbation was based on clinical documentation and may be subject to misclassification bias. Further differentiation between pneumonia, microbiologically confirmed and non-infective exacerbations was not performed due to the limited number of events, as additional subgrouping would have resulted in small and potentially unreliable groups with a risk of overinterpretation. Additionally, some potential confounders such as outpatient medication adherence and initial microbiological status could not be systemically monitored. Peri-interventional antibiotic prophylaxis was uniformly applied and may not reflect practices at other centers. Despite these limitations, the consistency and strength of the associations support the robustness of our findings.

In conclusion, elevated inflammatory markers, diabetes mellitus, prior exacerbation history and low BMI were independently associated with postprocedural exacerbations following BLVR with valves. These findings highlight the need for comprehensive patient assessment and may inform future strategies for personalized intervention and follow-up.

## Supplemental material

Supplemental material - Understanding exacerbation risk in BLVR: A logistic regression approach to complication prediction Supplemental material for Understanding exacerbation risk in BLVR: A logistic regression approach to complication prediction by Johannes Wienker, Kaid Darwiche, Rüdiger Karpf-Wissel, Erik Büscher, Johannes Haubold, David Kersting, Hubertus Hautzel, Lukas van de Sand, Aymen Kassem, Kristin Mersmann, Christian Taube, Marcel Opitz and Marc Struß in Chronic respiratory disease

## Data Availability

The datasets generated and analyzed during the current study are available from the corresponding authors on reasonable request.*
