# Supplementary material for: Understanding exacerbation risk in BLVR: A logistic regression approach to complication prediction
Source: Chron Respir Dis. 2026 Apr 22;23:14799731261443319. doi: 10.1177/14799731261443319 (PMC13111872; doi:10.1177/14799731261443319)
Supplement: Supplemental material - Understanding exacerbation risk in BLVR: A logistic regression approach to complication prediction [file sj-pdf-1-crd-10.1177_14799731261443319.pdf]

Table S1. Microbiological analysis

Specimen	Number	
	**Exacerbation**	**Pneumonia**
Citrobacter koseri	1	-
Escherichia coli	3	-
Klebsiella pneumoniae	2	1
Moraxella catarrhalis	2	-
Proteus mirabilis	2	1
Pseudomonas aerguinosa	1	2
Serratia marcescens	2	-
Staphylococcus aureus	3	-
Stenotrophomonas maltophilia	2	-
Streptococcus pneumoniae	-	2

Values are numbers of analyzed microbial specimen.
